# Biomarkers of Aging and Relevant Evaluation Techniques: A Comprehensive Review

**DOI:** 10.14336/AD.2023.00808-1

**Published:** 2024-05-07

**Authors:** Xue Tao, Ziman Zhu, Liguo Wang, Chunlin Li, Liwei Sun, Wei Wang, Weijun Gong

**Affiliations:** ^1^Department of Research, Beijing Rehabilitation Hospital, Capital Medical University, Beijing, China.; ^2^Beijing Rehabilitation Medicine Academy, Capital Medical University, Beijing, China.; ^3^Key Laboratory of Protein Sciences, School of Pharmaceutical Sciences, Tsinghua University, Beijing, China.; ^4^School of Biomedical Engineering, Capital Medical University, Beijing, China.; ^5^Beijing Key Laboratory of Fundamental Research on Biomechanics in Clinical Application, Capital Medical University, Beijing, China.; ^6^Department of Rehabilitation Radiology, Beijing Rehabilitation Hospital, Capital Medical University, Beijing, China.; ^7^Department of Neurological Rehabilitation, Beijing Rehabilitation Hospital, Capital Medical University, Beijing, China.

**Keywords:** aging, biomarkers, senescence, epigenetic modification, telomere

## Abstract

The risk of developing chronic illnesses and disabilities is increasing with age. To predict and prevent aging, biomarkers relevant to the aging process must be identified. This paper reviews the known molecular, cellular, and physiological biomarkers of aging. Moreover, we discuss the currently available technologies for identifying these biomarkers, and their applications and potential in aging research. We hope that this review will stimulate further research and innovation in this emerging and fast-growing field.

## 1. Introduction

Owing to advances in public health and medicine, population aging has become a severe challenge worldwide. In 2020, individuals aged 60 years and above outnumbered those aged less than 5 years. In the current trajectory, the share of the global population over the age of 60 will almost double from 12% to 22% between 2015 to 2050 [1]. Simultaneously, human life expectancy has been increasing, from an average of 67 years in 2000 to 71 years in 2015 [2]. Aging affects everyone and has substantial implications for the evolution of a nation's economy over time.

Aging is a gradual degenerative condition characterized by declining tissue stem cell reserves, shrinking organs, aging appearance, reduced life expectancy, decreased capacity to handle stress and injury, and the need for regeneration [3]. It is accompanied by organ damage, metabolic dysfunction, decreased bone density, matrix changes, tissue inflammation, and cell aging, the modifications of which reflect potential molecular alterations in nutrient perception, protein balance, mitochondria, intercellular interactions, DNA repair, and epigenetics [4], or the influence of external factors such as chemotherapy, smoking, radiation, high-fat diets, and other lifestyle or environmental factors [5]. These factors increase the risk of age-related diseases and mortality. Additionally, aging is a key risk factor for many diseases and disorders, including diabetes, osteoarthritis, Alzheimer’s disease (AD), cancer, and cardiovascular diseases [6, 7]. Without new models of medicine and healthcare, chronic diseases, which already have unfavorable social and economic repercussions, will continue to have an unsustainable impact globally. Fundamental studies on the mechanisms of aging and methods for reducing its consequences have been stimulated by the connection between aging and these disorders.

The search for reliable aging biomarkers is urgent, as they could enable better aging prediction, faster discovery of anti-aging strategies, and improved intervention and prognosis for age-related diseases. Furthermore, the discovery of reliable aging biomarkers may have substantial effects on preventing age-related ailments. For example, the development of neurofibrillary tangles is preceded by the concentration of senescent cells, indicating that senescent cells may affect how tangles form [8]. Furthermore, age-dependent AD pathogenesis might be mediated by molecular networks of gene regulatory elements, which could provide new therapeutic targets for AD treatment [9]. The search for precise and adaptable biomarkers of aging has benefited from the work of numerous scientists over the years. To identify and verify age indicators or advance basic gerontological studies and clinical translational research, the mechanisms of aging and its correlation with diseases must be clearly understood [10]. However, we must acknowledge the heterogeneity of aging paths, the causes and complexity of aging. To date, no reliable and independent aging biomarkers are available that can precisely depict an individual's aging condition or forecast the aging process and life span.

This paper reviews the current research hotspots, identifies cellular, molecular, and physiological biomarkers, and systematically evaluates the technologies and methods involving aging biomarkers. This review aims to assist readers in understanding concepts better, achieve insights into the effectiveness and limitations of existing studies and appreciate more vividly the application prospects and potential development trends of aging biomarkers.

## 2. Importance and Characteristics of Aging Biomarkers

### 2.1. Definition of aging biomarkers and their role in assessing biological age

Aging biomarkers are physiological and molecular indicators of age-related structural or functional degeneration at the fuselage, organ, tissue, cellular, and subcellular levels. It can be used to monitor and analyze the biological changes associated with aging, and to anticipate the progression of organ aging to disease. In the study of aging, three fundamental issues, including a person’s actual age, the reason for aging, and the possibility of healthy aging are explored. Aging biomarkers help establish biological age (BA). This is the basis and prerequisite for an in-depth aging assessment, early warning, and intervention for age-related diseases. Therefore, systematic research on aging biomarkers is important to promote the development of basic and translational medicine for aging and to help enhance the health of the elderly.

### 2.2. Key characteristics of effective aging biomarkers

The American Federation for Aging Research (AFAR) recommended the following criteria for reliable and advantageous biomarkers of aging [11]: (1) It must forecast age-related physiological, cognitive, and physical function of a person. That is, the BA must be determined, which more correctly characterizes one’s physical state than the chronological age (CA) and anticipates the future incidence of age-related diseases. (2) It must be testable and harmless to the test subject. Moreover, it must be technically straightforward for the vast majority of clinical laboratories to execute the test precisely and reliably without using any specialist tools or technologies. (3) It must be valid in both human beings and experimental animals, because experiments prior to clinical testing are always performed on non-human subjects. Therefore, in addition to avoiding discomfort or tension for the patients, the usage of biomarkers should be as simple and affordable as possible.

Recently, a systematic review suggested that a biometric measure for qualifying aging must be specific, systemic, and serviceable [12]. (1) Specificity: Aging is heterogeneous, not only in individuals of the same species, but also among different tissues and organs of the same individual. Therefore, different biomarkers will be required to evaluate the age of each organ in an organism. Similarly, each biomarker must capture one-of-a-kind aging signals of the organ being investigated. In addition, biomarkers of aging should predict the likelihood of development of illness, which necessitates a particular threshold marking the shift from physiological aging to pathological diseases. (2) Systemic: Aging affects each organ and system in the body, and changes in one organ may cause compensation or feedback in the whole body, so markers interact with each other and are not independent of each other. Biomarkers should be obtained from a variety of sources to accurately represent the systemic changes that occur with aging. (3) Serviceability: Biomarkers gathered using non-invasive techniques are ideal for translation into clinical applications. Given that aging is a process that involves gradual deterioration and requires longitudinal studies over a prolonged period, non-invasive techniques have become the preferred modality for detection.

Carlos et al. also proposed several criteria that must be applied to each aging biomarker. However, relative to the aforementioned characterization, they proposed a complementary one: markers that accelerate aging can be highlighted experimentally, and intervention in the aging biomarker could potentially decelerate, halt, or even reverse aging [13]. The disparity between BA and CA may serve as a basis for determining the extent to which certain markers contribute to aging. That is, objective quantification of the morphological and functional deterioration that affects aging organisms is the key to successfully assessing BA, both in laboratory animals and humans.

### 2.3. Importance of longitudinal studies for evaluating aging biomarkers

Aging is a pervasive process that results from a mix of elements ranging from hereditary factors to environmental influences. Extensive cross-sectional studies have been conducted on aging biomarkers, and the molecular changes accompanying human aging must also be studied to achieve a deeper understanding of the mechanisms of aging, because longitudinal studies are fundamental to understanding how the environment shapes the aging phenotype and influences the development of aging. Morgan et al. noted that aging gives rise to variable degrees of DNA methylation (DNAm) alterations in mammals and is considered to facilitate the development of age-related diseases [14]. A distinct DNAm age can be calculated by analyzing the methylation status in various tissues. This DNAm age correlated well with CA, which may be determined with relative precision within a few years [15]. In addition to epigenetic markers, some clinical indicators such as glycated hemoglobin and insulin-like growth factor-1 shift with advancing age [16]. This indicates that aging is accelerated when people check for important marker abnormalities, with a concomitant increase in the prevalence of associated metabolic or cardiovascular diseases. In another longitudinal study, the authors identified individual markers of aging that changed in short periods of two to three years. Moreover, they established many forms of aging patterns that may occur in different individuals according to the kinds of molecular pathways that can be altered throughout the course of one’s lifetime in a particular individual, thereby offering molecular analysis of each individual that could be improved at the individual level through selective interventions or lifestyle changes [17]. Therefore, over time, aging biomarkers could potentially control aging at an individual level. In conclusion, longitudinal studies will help us gain a better insight into the effects of aging on specific functions of organs and systems and help us understand the relevant features of the function and structure of each system during different physiological processes, as well as their changes during the individual life cycle. Combined interventions at a specific time of aging may prevent the emergence of age-related diseases and improve the quality of life, even prolonging one’s healthy life expectancy.

## 3. Molecular Aging Biomarkers

### 3.1. Telomere length and telomerase activity as indicators of cellular aging

**Figure 1.**
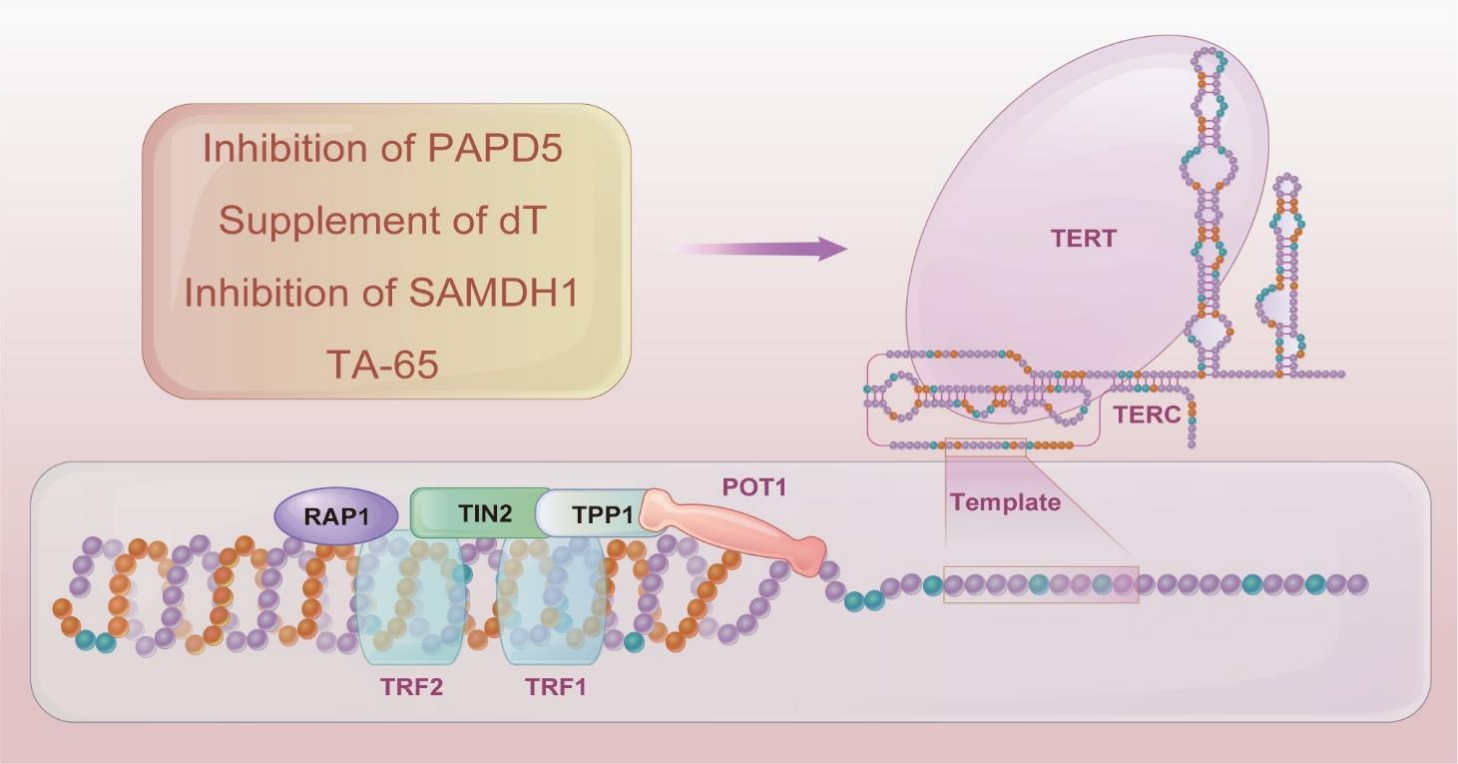
**Structure of the vertebrate telomere/telomerase complex**. Inhibition of PAPD5, supplement of dT, inhibition of SAMDH1, TA-65, etc. were reported to promote telomere recovery. POT1, protection of telomeres 1; TRF1, telomeric repeat binding factor 1; TPP1, telomere protection protein 1; RAP1, TERF2-interacting protein; TRF2, telomeric repeat binding factor 2; TIN2, TERF1-interacting nuclear factor 2.

Telomere length (TL) and telomerase activity (TA) have been established as essential indicators of aging. To evaluate the drop in TL with age, cross-sectional data over the period 1999-2022 on 7826 people undergoing TL measurement from the National Health and Nutrition Examination Survey of the US population were examined, and the results revealed that TL became shorter with age [18]. Age-related TL changes have also been observed in circulating peripheral blood mononuclear cells of three male rat species: Brown Norway, Sprague-Dawley, and Fischer 344 [19]. Telomeres are DNA-protein constructions at the ends of chromosomes that consist of multicomponent protein complexes including shelterin and short tandem DNA repeats [20]. Due to issues with terminal replication, telomeres shorten throughout cell division when the DNA replication machinery fails to finish synthesis at the tip of linear chromosomes [21]. However, the loss of telomeres can be offset by telomerase [22]. As shown in Figure 1, telomerase is a ribonucleoprotein complex composed of a telomerase reverse transcriptase (TERT) catalytic subunit, which copies the telomerase RNA component (TERC) and synthesizes new telomere repeats [3]. The systematic knockout of telomerase subunits results in a shorter lifetime, rapid organ failure, and a TL reduction [23-26]. Overexpression of TERT in mTert^-/-^ cells reversed the accelerated senescence and transformation phenotypes caused by the removal or downregulation of TERT expression using CRISPR/Cas9 or shRNA [27]. Similarly, telomerase reactivation in TERT-ER mice extended telomeres, reduced DNA damage signaling, and reversed degenerative phenotypes in multiple organs [28]. Animal models in which telomerase genes are absent or inducible have helped researchers establish links among telomeres, telomerase, aging and related dysfunctions, thereby confirming the crucial role of telomeres in aging.

Consequently, intervening in the biological processes associated with telomeres or telomerase to slow aging or improve age-related dysfunction must be studied. An in-depth understanding of telomerase-regulated molecular networks and telomerase-activated regeneration in prematurely aged mice has stimulated interest in developing anti-aging drugs that activate TERT expression. In the embryonic fibroblasts of haploid defective mice, a small molecule telomerase activator can lengthen the average telomeres, lower the fraction of extremely short telomeres, and lessen DNA damage [29]. Inhibition of PAPD5, an oligo-adenylation and TERC-stabilizing polymerase, could restore TL in pluripotent stem cells induced in patients with congenital dyskeratosis [30]. In addition, genome-wide thymidine (dT) nucleotide metabolism was identified as a constraint in the maintenance of human telomeres via CRISPR/Cas9 functional TL screening. When telomere anomalies are inherited, SAMHD1 inhibition or dT supplementation may help regenerate the telomeres of induced pluripotent stem cells [31]. Sirtuin 6 (SIRT6) is closely associated with telomere integrity maintenance. It deacetylates telomeres during the S phase to stabilize specific telomerase enzymes and prevent abnormal loss of telomere sequences; additionally, it promotes chromatin depolymerization of damaged telomeres, which substantially affects the control of telomere movement following injury [32]. To explore whether short telomeres are irreversibly damaged after reestablishment of telomerase activity, the researchers crossbred Terc^+/-^ mice with late-stage telomerase-deficient Terc^-/-^ mice that had been reintroduced to telomerase, resulting in offspring with identifiable telomeres and free of chromosomal instability, and unaffected by early aging [33]. Various aspects remain unclear, such as the accuracy of TL, TA, and TERC measurement methods, whether TL and TA can be employed as direct markers of age-related disorders and aging in clinical trials, the control mechanism of telomerase expression and activity, and the mechanism of action of telomerase activators. Therefore, further studies are necessary.

### 3.2. Epigenetic modifications

Nearly a decade ago, numerous CpG sites in the human genome have shown that methylation rates increased or decreased over time [34]. Epigenetic modifications such as DNAm, histone acetylation, phosphorylation, and ubiquitination are emerging areas of aging biomarker research. These modifications regulate transcription and X-chromosome inactivation by altering promoters, enhancers, and genes [35, 36]. For instance, aging kidneys show reduced expression of the anti-aging factors KLOTHO and Nrf2 (nuclear factor erythroid 2 - related factor 2), along with increased hypermethylation of the Nrf2/KLOTHO gene promoters and DNA methyltransferase (DNMT) 1/31/3b. SGI-1072 (a DNMT inhibitor) reversed these changes [37]. Therefore, epigenetic modifications are thought to be correlated with CA at the molecular level and are promising candidates for quantifying biological aging rate, measuring lifespan, or intervening in rejuvenation [38]. Belsky et al. demonstrated that the rate of biological aging can be gauged using a DNA blood test [39]. Ran et al. have detailed the properties, differences, and applications of epigenetic clocks in aging research [40]. Specifically, several classic epigenetic clocks, such as DNA PhenoAge, DNA GrimAge, Hannum's clock, Dunedin PACE, and Horvath's clock, have been proposed as effective techniques to promote healthy aging and as aging biomarkers and have also been employed as useful tools to assess the effectiveness of age-reversing interventions [40, 41]. However, differences in statistical methods, sample sizes, and tissue types result in unique calibration methods for each epigenetic clock. Existing epigenetic clocks vary not only in CpGs and Illumina arrays, but also in tissue origin and ethnicity, which restricts their applications [42]. For example, most clocks were trained using only whole blood samples, whereas two clocks contained multiple tissue samples (Table 1).

**Table 1:** Summary of recent existing 12 epigenetic clocks in human samples.

First author, Year	# of CpGs	Illumina array	# of subjects	Age	Tissues used in training	Training phenotype	Regression	Prediction accurady	Ref.
**Bocklandt, S., 2011**	1	27K	68	21-55	Saliva	Chronological age	lasso penalized regression	0.77	[245]
**Garagnani, P., 2012**	1	450K	64	9-83	Whole blood	Chronological age	Spearman	0.92	[246]
**Hannum, G., 2013**	71	450K	482	19-101	Whole blood	Chronological age	penalized regression model	0.905	[247]
**Horvath, S., 2013**	353	27K, 450K	7844	0-100	51 healthy tissues and cell types	Chronological age	penalized regression model (elastic net)	0.960	[34]
**Weidner, C.I., 2014**	3	27K	575	0-78	Whole blood	Chronological age	multivariate regression model	0.87	[248]
**Lin, Q., 2015**	99	450K	656	19-101	Whole blood	Mortality	multivariate Cox regression	-	[249]
**Vidal-Bralo, L., 2016**	8	27K	390	20-	Whole blood	Chronological age	forward stepwise linear regression	0.68	[250]
**Yang Z., 2016**	385	450K	650	0, 80-	Whole blood	Chronological age	linear regression	-	[44]
**Zhang Y., 2017**	10	450K	1000	50-75	Whole blood	Mortality	linear combination of LASSO regression	-	[43]
**Levine, M., 2018**	513	27K,450K, EPIC	9926	0-100	Whole blood	Phenotypic Age	cox penalized regression model	-	[45]
**Horvath, S., 2018**	391	450K, EPIC	896	0-94	Skin and blood	Chronological age	elastic net regression	-	[251]
**Lu, 2019**	1030	450K, EPIC	6935	46-78	Whole blood	lifespan	elastic net Cox regression model	-	[252]

In addition, while most are developed as “predictors of CA”, three are trained using different phenotypes that depict how aging affects different aspects of an individual’s health. Zhang et al. created a clock to predict mortality from all causes [43], and Yang et al. trained the clock to approximate mitosis rates [44]. The Levine et al. clock was designed to simulate a multi-system clinical measure of aging that closely correlates with age, but separates people of similar ages based on morbidity and mortality risk [45].

Liu et al. developed a new clock based on 11 classical clocks that were decomposed into submodules and recombined into more powerful epigenetic aging measures. The clock showed considerably higher ability to detect senescent cell states, such as cancer and aging, and forecast mortality risk based on DNAm in blood [46]. Recently, epigenetic clocks have been used in the randomized clinical trials to validate the effects of an interventions [47]. A series of subsequent clinical studies further emphasized the importance of studying potential changes in DNAm levels during aging [48, 49]. In general, epigenetic age prediction is easier and more effective. Packages for the epigenetic clock are widely accessible, some of which require only the methylation values of a few CpG sites to accurately predict age [50]. However, additional assessments must be conducted before a high degree of confidence can be placed in these reports. In addition to building more accurate time clocks, the precise processes through which the epigenome influences aging remain unclear.

### 3.3. Genetic variants associated with aging and longevity

The role of genetic variants (GVs) in aging is the subject of ongoing debate. GVs, which include single nucleotide polymorphisms (SNPs) in structural variants, such as copy number variants (CNVs) in the human genome, contribute to population diversity and influence the study of the genetic effects of complex traits and disease vulnerability. According to genome-wide association studies, various complex diseases and traits have been linked to numerous GVs according to genome-wide association studies (GWAS) [51]. In both animal models and human populations, certain GVs affect age-related phenotypes. For example, knock-in mice carrying an *Lrp6* variant exhibited age-dependent structural and functional synaptic defects [52]. Fifty-two aging variables were examined in a phenotype-wide association analysis of 379,758 UK Biobank participants from European heritage sites to confirm the association between SH2B3 missense variants and aging [53]. Another gene involved in aging encodes thioredoxin reductase (TXNRD), an antioxidant enzyme that protects organisms from oxidative stress. Variations in TXNRDI have been correlated with physical and cognitive functions in older people [54], which was validated in a cohort study from southern Italy [55]. Age-dependent obesity is a major public health problem that may be influenced by GVs. To identify GVs that show differential effects on age-dependent obesity, Ju et al. conducted a GWAS on 355,335 UK Biobank participants, stratified the analysis of five obesity-related phenotypes, and conducted t-statistics. Five significant lead SNPs were identified: rs9861311 and rs429358 for body fat percentage, rs2258461 for body mass index, rs145500243 for waist circumference, and rs2870099 for waist-to-hip ratio [56]. Notably, rs429358, located in APOE, is also associated with various age-related diseases such as cognitive decline, coronary artery disease, and age-related macular disease [57, 58]. However, the contributions of these genes to the aging process are limited. After thorough cleansing and verification, Jonna et al. studied 86 million profiles from openly accessible Internet genealogical data and obtained a single-family tree that included 13 million people. These data were used to estimate the heritability of human longevity, and the results showed that genes play a marginal role in longevity [59], similar to the result of another study published in the same year [60]. According to some studies, centenarians may have fewer detrimental alleles, such as rare non-synonymous SNP variants, while others show that centenarians share similar risk GVs for major diseases to those in the younger population [61, 62]. Ukraintseva et al. explained this discrepancy by considering the conditional and trade-off-like effects of various biological mechanisms, including the antagonistic effects of genes on various health conditions and age-specific mortality susceptibility, interactions between genes and the environment, and other factors [63, 64]. This suggests that when evaluating the aging status or implementing interventions based on GVs, we must also consider the trade-off-like effects of GVs that may be caused by diseases to provide personalized treatment or assessment and reduce all-cause mortality.

## 4. Cellular Aging Biomarkers

### 4.1. Cellular senescence markers and associated phenotypic changes

**Figure 2.**
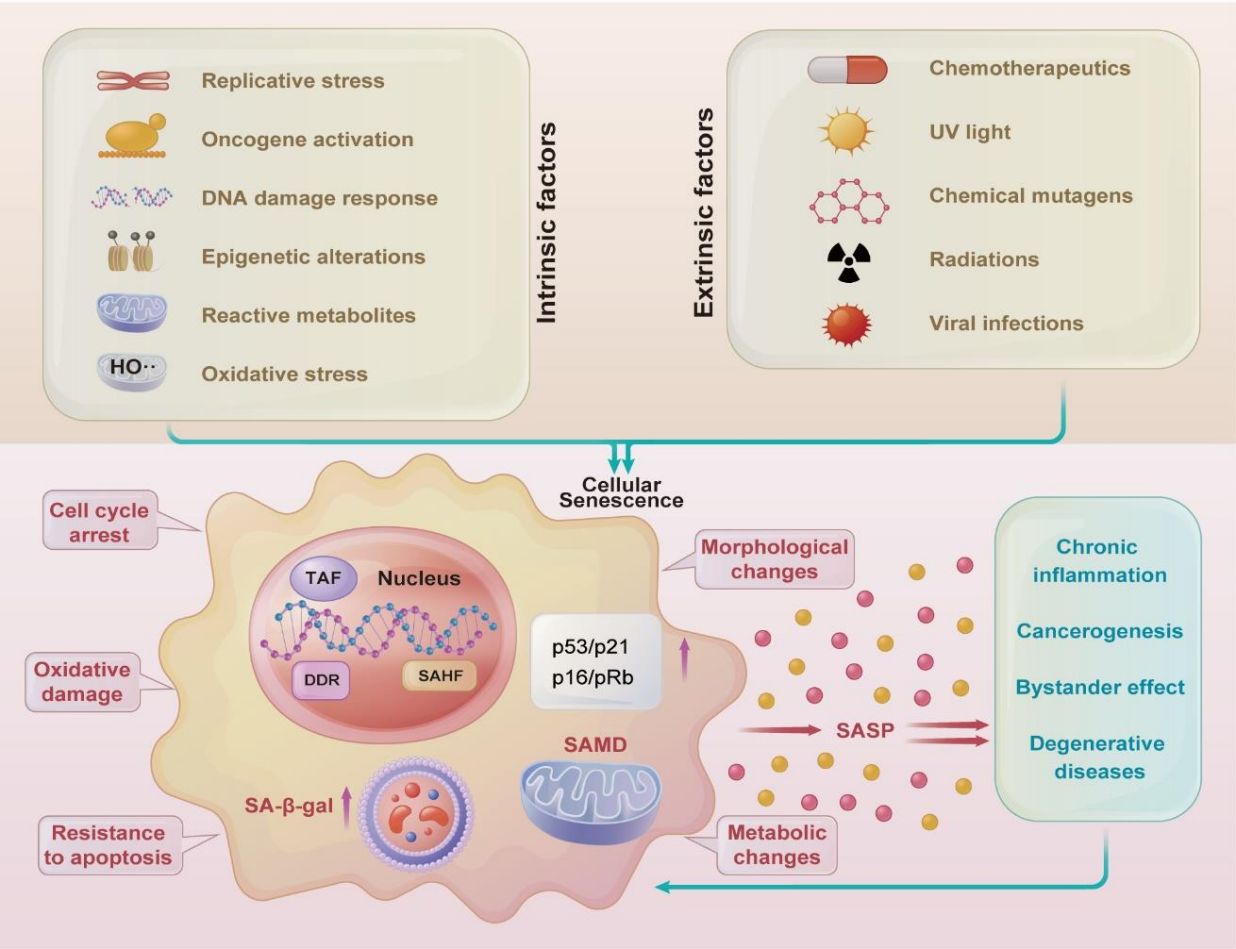
**Various stimuli can induce cellular senescence, and the senescence-associated secratory phenotype (SASP) can cause a series of negative consequences**. SAHF, senescence-associated heterochromatin foci; SA-β-gal, senescence-associated β-galactosidase; TAF, telomere-associated foci; SAMD, senescence-associated mitochondrial dysfunction; DDR, DNA damage response.

Cellular senescence was first described in 1961, when Hayflick et al. discovered that diploid fibroblast lines ceased to divide after a 40-60 fold multiplication [65]. Generally, cellular senescence is characterized by morphological flattening, enlargement, and irregularly shaped bodies, which occur because the cytoskeleton rearranges and involves vimentin intermediate fibers and microtubules [66]. As shown in Figure 2, senescent cells exhibit drastic changes in chromatin structure and gene expression [66]. Even under optimal growth conditions or mitogenic stimuli, somatic cells that enter senescence, stop proliferating and enter a long-term stable phase of growth arrest while maintaining their metabolic capacity and viability, and even resisting apoptosis [67]. The resistance to apoptosis exhibited by senescent cells may be related to the activation of cell survival mechanism, including those of the anti-apoptotic protein BCL-2 family [68]. The fate of cells is determined by the duration and intensity of the initial stimulations, and the nature and cell type of the damage. Moreover, cellular senescence has been suggested to serve as a defense mechanism to stop damaged cells from spreading [69]. Cellular senescence has also been implicated in aging and age-related diseases. The senescence-associated secretory phenotype (SASP), which refers to substances secreted by senescent cells, influences other cells and tissues. The accumulation of SASP components and senescent cells can impair tissue regeneration, promote chronic inflammation, and contribute to aging.

Various markers, such as p16, p21, and senescence-associated -galactosidase (SA-gal), have been used to differentiate senescent cells from healthy, non-proliferating cells; however, none of these markers are universal indicators of cellular senescence [70]. SA-β-gal is the mostly used marker for cellular senescence, the activity of which is upregulated at pH 6, which is the pH of intra-lysosome of senescence cells [71]. Deng et al. reported a novel precursor drug development strategy based on this marker; namely, the precursor drug SSK1 was designed to be specifically cleaved into cytotoxic substances by lysosome-gal in senescent cells and to induce apoptosis, thereby eliminating them [72]. However, SA-gal activity has also been observed in lysosome-active cells such as macrophages in a variety of post-mitotic cells, including neurons, and even in the early stages of embryonic development [73]. Authors observed a delay between senescence entry and SA-β-gal derived staining [74]. Moreover, the activity of endogenous SA-β-gal is also observed in confluent non-transformed fibroblast cultures [73, 75]. All these studies suggest that SA-β-gal as a biomarker for cellular senescence is lacking in specificity and sensitivity. Cell cycle arrest can be induced by the cyclin-dependent kinase inhibitors p16 and p21. The main tumor suppressor protein p53 typically controls these cells, and its increased expression can serve as a marker of cellular senescence [76]. Satotaka et al. established a mouse model p16-Cre^ERT2^- tdTomato analyzed the single-cell properties of p16^high^ cells. They found that p16^high^ cells positive for tdTomato were detectable in all organs and accumulated with age [77]. Xue et al. revealed that ADAR1 controls the expression of p16^INK4a^ through post-transcriptional regulation and modulates cellular senescence, independent of RNA-editing [78]. However, p16^INK4a^ staining of keratinocyte subsets in wound margin tissue was also evident in patients undergoing selective amputation due to severe limb ischemia [79]. Hes1 was found to differentially regulate the proliferation of neural stem cells via p21 [80]. Laminin is an extracellular matrix glycoprotein that defines the shape of the nucleus, influences DNA replication and repair, regulates gene expression, participates in stress responses, and is involved in cell cycle processes [81]. Consequently, lamins, which are nuclear envelope proteins that interact with laminin, are involved in cellular senescence. Lamin B1, a nuclear layer factor down-regulated in senescent cells, is involved in the regulation of subtelomere genes [82]. The knockdown of METTL14 reduced the m^6^A level of the lamin B receptor, leading to instability of the lamin B receptor mRNA, which in turn leads to cellular senescence [83].

### 4.2. Mitochondrial function and oxidative stress markers

Reactive oxygen species (ROS) and energy are produced by the mitochondria and are essential for cell metabolism. However, cellular components such as proteins, lipids, and DNA can sustain oxidative damage from ROS, implicating mitochondria as a major source of age-related damage in cells and tissues [84]. Miquel et al. were the first to link aging and mitochondria, and observed that moderate temperature reduction extended the lifespan of poikilotherms by lowering their metabolic rate [85]. They also noted that large animals tended to exhibit lower rates of basal metabolism and lived longer than smaller animals. They suggested that a lower metabolic rate limited ROS generation and mitochondrial oxidative damage, thereby delaying aging [85]. According to the mitochondrial free radical theory of aging (MFRTA), aging results from the cumulative oxidative damage caused by an imbalance between antioxidant defenses and ROS generation in cells and tissues [86]. According to this theory, mitochondria, particularly mitochondrial DNA (mtDNA), are the main sites of ROS generation and important targets of ROS-induced damage. Mutations in the mtDNA can impair mitochondrial function and increase ROS production, thereby creating a vicious cycle of oxidative stress and aging [87]. However, mtDNA exists in multiple copies and a single mtDNA mutation is unlikely to cause functional abnormalities. This abnormality has a threshold, and ROS-induced mitochondrial dysfunction gradually accumulates. Moreover, defects in nuclear replication can affect mitochondrial energy production [88]. Studies over the last century have established a relationship between mitochondria and oxidative stress during aging, indicating that mitochondrial function, ROS levels, antioxidant capacity, and tissue repair ability are important factors in aging. However, whether mitochondria and antioxidants are the cause or consequences of aging remains unclear. Recently, these factors were shown to be associated with longevity at the cellular level but required further validation at the animal level. Furthermore, the measurement of tissue oxidative damage alone seems to provide more conclusive evidence than testing for mitochondrial ROS and other oxidative or antioxidant factors. Specific amino acids or lipids may serve as markers for aging [89]. For example, methionine or cysteine may be sensitive to oxidation, and therefore scavenge oxidized components and participate in oxidized tissue repair [90]. DNA damage caused by ROS can produce DNA damage markers, which can be assessed by evaluating DNA damage and DNA repair mechanisms. These markers can also be used to evaluate aging.

Recently, the role of mitochondria in oxidative stress-induced aging has been investigated. De Lucia et al. revealed that serum mitochondrial activity did not differ between older and younger people [91]. Van et al. reported that mice with reduced expression of superoxide dismutase (SOD) had increased DNA damage and tumor incidence but did not show accelerated aging or reduced lifespan compared with the control group [92]. Furthermore, previous studies have considered a decline in mitochondrial function as a marker of aging. However, Liu et al. demonstrated that disrupting oxidative respiratory chain function without causing cell death can not only impair mitochondrial function, but also alter the growth rate and extend lifespan in worms, flies, and mice [93]. Therefore, the assessment of mitochondrial function alone may not explain aging. In conclusion, mitochondrial damage, DNA damage, and oxidative stress are interrelated factors that influence aging and longevity in various organisms. However, the exact mechanisms and interactions between these factors have not been fully elucidated and require further investigation.

### 4.3. Markers of inflammation and immune senescence

Innate and adaptive immune responses that become dysfunctional with age weaken pathogen defenses and increase mortality and morbidity rates. This phenomenon, often termed immune aging, encompasses an abundance of memory T cells, a decrease in antigen-reactive capability, alterations in calcium-mediated signaling, damage and atrophy of the thymus, and persistent low-grade inflammation driven by the SASP [94]. One aspect of immune aging is the progressive deterioration of the innate immunity mediated by phagocytes. The magnitude and timing of the type I interferon (INF) response determines susceptibility to viral infection. In older adults, the production of type I INF by circulating plasmacytoid dendritic cells (pDC) is diminished and delayed [95]. Immune cells such as NK cells and macrophages can be recruited and activated by type I IFN, whose reduction affects multiple levels of the innate immune response during aging [96]. Aged alveolar macrophages exhibit a reduced ability to phagocytose neutrophils, leading to prolonged neutrophil retention and tissue damage [97]. Aging also leads to an increase in the number of bone marrow-derived suppressor cells (MDSCs), which have the ability to restrict T-cell activity and thus contribute to the pathogenesis of a number of illnesses, including infectious diseases and cancer [98].

Even in mouse models lacking exposure to infections, adaptive immune responses that are predominantly driven by T and B cells were less effective with age. Aging reduces the quality and level of B-cell responses to infection, leading to lower antibody production and affinity [99]. In mouse models with accelerated immune aging and natural aging, spleen B cells showed increased expression of p16^INK4a^ and p21^Cip1^ but limited expression of SASP compared to other immune subtypes [100]. A subpopulation of B cells called age-related B cells (ABC) increased with age in mice [101]. The phenotype of these cells was defined as B220CD19^+^, but they were deficient in the mature B cell markers, CD21 and CD23. Additionally, CD11c and T-bet, which drive cells toward a pro-inflammatory phenotype, are also expressed in ABC. Through targeted death of infected cells, CD8t cytotoxic. T cells aid in pathogen removal. High levels of p38 and yH2AX, along with other age-related characteristics, including a decreased rate of proliferation and shortened telomeres, are present in highly differentiated CD27^-^CD28^-^CD8^+^ T cells [102]. CD45RA^+^CD27^-^CD4^+^ T cells were suggested to be senescent T cells with reduced telomerase activity and proliferation, and constitutive p38 expression. With the reduced activation of T cell receptors (TCRS), senescent T cells express NK cell receptors. These cells may cause substantial tissue damage via NK cell receptors (NKRs) as opposed to T cell receptors because they express high levels of cytotoxic chemicals [103]. In a recent study that employed the SA-gal staining approach to detect “true” senescent immune cells in human aging, senescent circulating T cells, particularly CD8^+^ T cells, showed senescent gene expression characteristics and decreased proliferation potential. High SA-β-gal CD8^+^ cells, however, displayed a T-cell destiny that was distinct from that of T cells that were previously classified as senescent [104]. In addition, the expression of p16^INK4a^ in CD8^+^ T cells was associated with both BA and illness [105]. Dysfunctional T cells exhibited strong expression of the retrotransposon LINE-1, whose reduction can restore the function of the T cell effector. LINE-1 expression levels are crucial in controlling T cell effector function, quiescence, and failure, although LINE-1 is known to be increased in senescent cells, triggering a type 1 IFN response [106].

Many senescent cells exhibit a pro-inflammatory SASP, which involves the release of various chemokines and cytokines that modulate or attract immune cells [107]. The SASP may also include the secretion of metalloproteinases, microRNAs, reactive oxygen species, metabolites, and extracellular vesicles. Through the SASP, the build-up of senescent cells in aging organs causes regional and systemic inflammation, impairs stem cell function, and promotes tissue degeneration [108]. Bora et al. analyzed Tabula Muris Senis data and identified 10 age-related genes, among which interleukin 1β (IL1b) was particularly enriched in almost all the Kupffer cells in older mice compared with approximately 37% in younger mice [109]. Another study showed that the deficiency of Ercc1 (part of the Ercc1XPF endonuclidene enzyme, which is crucial for various DNA repair processes, especially in hematopoietic cells) impaired the repair of DNA damage, accelerated the increase of senescent cells and endogenous oxidative damage in various tissues in mice [110]. The absolute number of pDCs in the intestinal epithelium of aged mice was substantially reduced and the expression of the inflammatory chemokine CCL25 changed with age [111]. Moreover, senescence and SASP production in several organs were induced by the adoptive transfer of spleen cells from old WT mice to young mice, which considerably decreased longevity [112]. According to these findings, DNA damage can cause immune cells to become more senescent as they age; subsequently, the secreted SASP factors can trigger secondary aging. The specific immune cell subpopulations that are responsible for senescence and systemic aging in lymphoid and non-lymphoid organs remain unclear. Conversely, a mouse model of accelerated aging and senescence was adoptively transferred with young immune cells, which resulted in a reduced number of senescent cells in many tissues, demonstrating the ability of young immune cells to eradicate SnCs that develop with age and disease.

## 5. Physiological Aging Biomarkers

### 5.1. Physical performance and functional decline assessments

**Figure 3.**
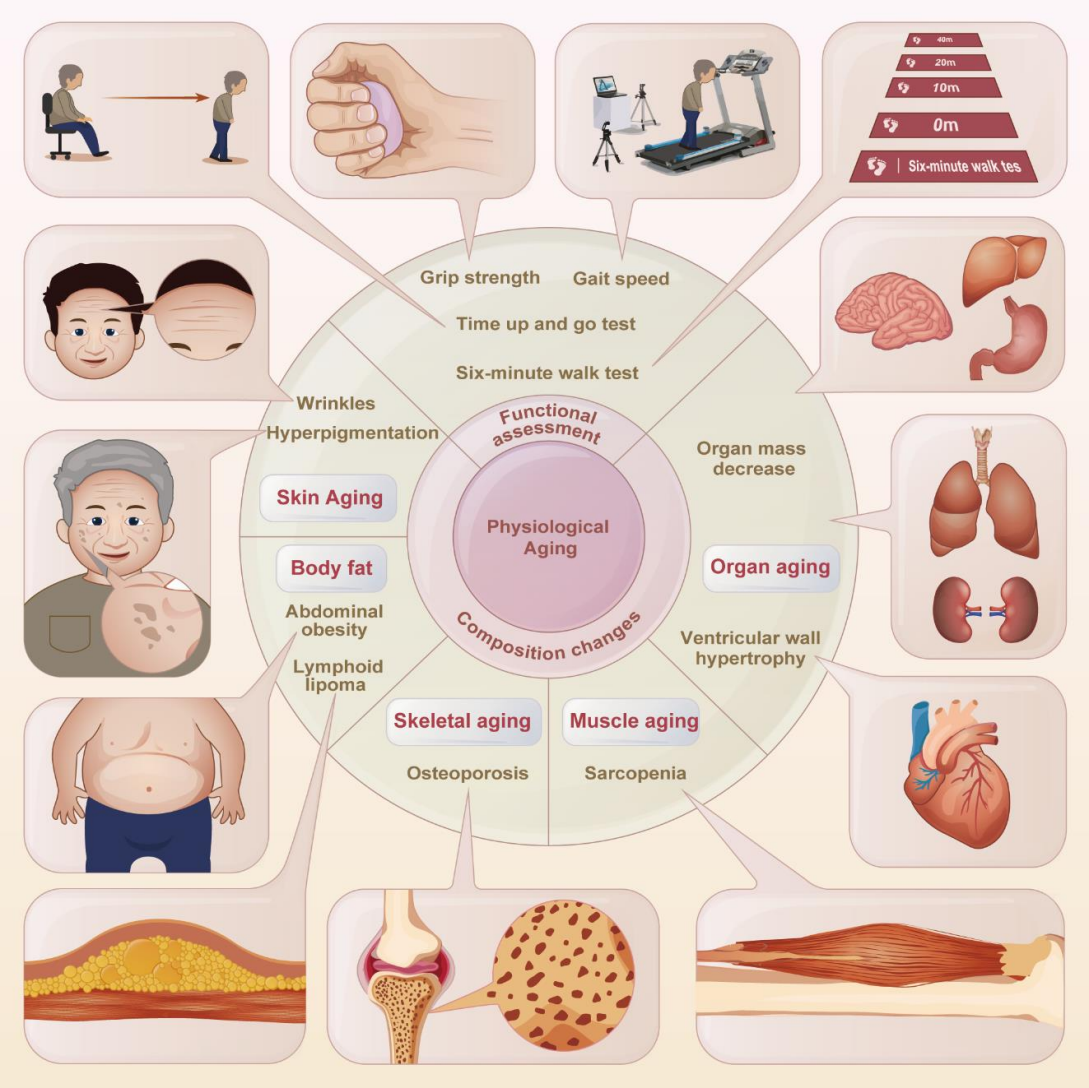
Physical performance and functional decline assessments.

Physical function metrics are vital for gauging present and future health. Physical functional assessments (Fig. 3) that are both objective and standardized have been created and are increasingly used in population research. Physical aging can be tracked using common functional measures, including the grip strength, gait speed, time up and go test, and six-minute walk test [113]. Many studies and systematic reviews conducted to evaluate the risk of subsequent disability have shown that elderly people who perform poorly on physical functional tests (e.g., weaker grip strength, slower walking speed, or poorer standing balance) are more likely to be functionally handicapped in the future [114, 115].

Aging is also linked to changes in physical composition such as the development of wrinkles and hyperpigmentation, increased body fat, reduction in organ mass, decline in muscle mass and strength, and osteoporosis. Skin aging is a readily observable sign of aging and a direct reflection of the body’s age. Wrinkles, particularly on the face, are the clearest indicator of skin aging. Histological evidence from aged, wrinkled skin shows a rough distribution and volume decrease of collagen and reduction in the number of elastic fibers in the dermis extracellular matrix components [116]. Changes in pigmentation are another indicator of skin aging. These spots often emerge in sun-exposed regions and are a consequence of increased pigment synthesis caused by exposure to ultraviolet radiation [117]. Higher body mass index (BMI) is a risk factor for diseases associated with aging, with a 30% rise in overall mortality for each 5 unit rise in BMI [118]. A study from the Nanyang Technological University in Singapore showed that higher levels of visceral fat, BMI, and reduced high-density lipoprotein (HDL) levels were causally associated with cognitive decline in Asian populations. Specifically, each additional 0.27 kilogram of visceral fat is comparable to an additional 0.7 years of cognitive aging [119]. Furthermore, the lipids are redistributed over age [120]. Excess fat also attacks the immune system, with the exception of accumulation in the liver and bone marrow. Lymph nodes undergo a process in which normal tissue is gradually replaced by adipose tissue, leading to lipomatosis [121]. Eventually, fat accumulation in the lymph nodes renders them non-functional. Skeletal muscle constitutes the majority of the total tissue mass. Age-related degeneration of the neuromuscular junction has been documented in both animal models and humans. During aging, synaptic transmission is impaired and muscle fibers lose innervation, resulting in degeneration and atrophy [122].

Muscle mass and strength become progressively weaker and muscle function decreases with muscle fiber atrophy after 40 years, which is known as sarcopenia [123]. Kim et al. reported that the grip strength, muscle mass, and endurance of 18-month-old C57BL/6J mice were substantially lower than those of 10-week-old mice, demonstrating an increasing incidence of sarcopenia in aging animals [124]. Similarly, compared to 10-month-old mice, 25-month-old mice showed substantially reduced hind limb muscle mass, muscle grip strength, maximal muscle strength, and daily activity capacity [125]. Aging affects all organ systems, but the trajectory of aging varies across organ systems and between individuals. Except for the heart, organ mass mostly decreases with age, resulting in impaired reserve capacity and limited reactivity to stress [12]. As aging progresses, bone resorption increases, resulting in reduced bone density [126]. Dual energy X-ray absorptiometry (DXA) is recommended by physicians in the US for men aged > 70 years and women aged > 65 years to test the bone mineral density (BMD) at the lumbar spine and femoral neck. An osteoporosis determination is made once the T-score is less than or equal to -2.5 [127]. On the other hand, both the C-telopeptide of type I collagen (CTX-I) and the N-terminal propeptide of type I procollagen (PINP) are indicated for use in clinical settings as indicators of bone production and resorption, but can only be used to support the diagnosis of osteoporosis [128].

### 5.2. Cardiovascular and metabolic biomarkers of aging

Age is one of the most powerful predictors of cardiovascular health and regular cardiovascular function is an important factor in determining whether a person will live a long and healthy life. As the human cardiovascular system is composed of many distinct cell types, aging may affect the biological activity of each cell type in the body. Cardiomyocytes and cardiovascular cells are especially prone to age-related dysfunction and failure, leading to structural damage; heart dysfunction, such as myocardial cell death, myocardial hypertrophy or fibrosis, reduced elastic fibers, and valve calcification, is also an indicator of cardiac aging. The direct result of these pathological features is cardiac conduction malfunction derangement and systolic-diastolic dysfunction [129, 130], which in severe cases leads to age-related heart failure [131]. Over the past 20 years, various biomarkers reflecting neuroendocrine activation, myocardial injury, cardiac remodeling, inflammation, and other pathophysiological alterations in heart failure have been identified [25], focusing on biomarkers active during cardiac load, myocardial injury, and matrix remodeling.

N-terminal pro-B-type natriuretic peptide (NT-proBNP) and B-type natriuretic peptide (BNP) in the natriuretic peptide family are the most important biomarkers of cardiac load and have the highest recommended category among all biomarkers of heart failure. While a correlation has not been observed, their clinical applications are comparable. For example, compared to BNP, NT-proBNP is more concentrated in peripheral blood and more stable in vitro. It has no biological activity, and aging and renal function have a greater impact. BNP/NT-proBNP testing is indicated for both the support and differential diagnosis of heart failure in individuals who are older and present with symptoms of the condition, when BNP ≥ 35 pg/ml or NT-proBNP ≥ 125 pg/ml is present for a long period of time, a more thorough examination is recommended [132].

Cardiac troponin T (cTnT) and cardiac troponin I (cTnl) are the most commonly used clinical biomarkers for assessing myocardial injury. The two biomarkers are different to a certain extent: the half-life of cTnT is 30 min longer than that of cTnl; cTnl is less affected by renal function compared to cTnT; cTnI is more specific to cardiac tissue than cTnT; and cTnT is also seen in neuromuscular diseases in addition to myocardial injury. High-sensitivity cTnI (hs-cTnI) can be used to independently predict the onset of heart failure; however, the highest predictive value for HF was obtained when hs-cTnI was paired with NT-proBNP [133]. Furthermore, hs-cTnT has also been found to be related to the incidence of heart failure in cohort studies, and its increase over time suggests the progression of myocardial injury, particularly in those with >50% elevation from baseline, together with an accompanying increase in the probability of mortality in the coming years [134].

Currently, soluble growth stimulation expressed gene 2 (sST2) and galectin-3 (Gal-3) are the most common indicators of cardiac stromal remodeling used in clinic. Multiple studies have demonstrated considerably higher levels of sST2 and Gal-3 in patients with HF than in those without HF, and the extent of this increase corresponds to the degree of diastolic dysfunction [135]. Various molecular signaling pathways are altered during cardiac aging. ROS accumulate in the aging heart and cause mitochondrial oxidative stress damage, which is associated with increased mtDNA mutations and deletions [136]. In contrast, cardiac autophagy decreases with age. Taneike et al. studied age-related changes in autophagy in the hearts of wild-type mice and found that autophagy markers, such as microtubule-associated protein 1 light chain 3-II (LC3-II), were decreased in 14- or 26-month-old mouse hearts compared with 10-week-old mice. mTOR plays an important role in controlling autophagy [137]. Aging has been shown to enhance mTOR phosphorylation in mouse hearts [138], while inhibition of mTOR prolongs mice lifespan [139].

### 5.3. Neurological and cognitive markers

The brain is the command center of the body. Together with the spinal cord, it constitutes the central nervous system, which innervates the entire body via the peripheral nervous system. The brain degenerates over time, which increases the risk of age-related neurodegenerative diseases. Morphological changes in the brain and pathological deposition of abnormal proteins such as amyloid-beta (Aβ), tau and alpha-synuclein [140], and alterations in physiological function, together affect human lifespan. Morphological changes in the brain include reduced brain volume, ventricular enlargement, cortical thinning, and white matter degradation caused by neuronal reduction, dendritic degeneration, demyelination, microglial activation, white matter lesions, and metabolic slowdown [141]. These multiple alterations in the brain may form the basis of age-related cognitive decline. Integrating several studies on whole-brain volume changes in humans over a limited age range reveals that such changes occur throughout the lifespan, with the brain tissue beginning to decrease steadily after 35 years and brain volume loss steadily exceeding 0.5% per year over 60 years of age [142]. Unlike physiological aging, neurodegenerative diseases show different patterns of atrophy, such as medial temporal lobe atrophy, which is a hallmark of AD [143]. A reduction in neuronal volume rather than number, and a decrease in dendritic spines are believed to be the primary causes of age-related brain atrophy [144]. These morphological changes in neurons cause synaptic dysfunction and impair neurotransmitter signaling, which in turn affects cognitive function [145]. Synaptic density in the CA1 area of the hippocampus is lower in aged rats than in younger animals, and the spatial memory capacity decreases [146]. Aged rats have reduced spatial memory capacity compared to younger rats, and histological examination has revealed lower synaptic density in the CA1 region of the hippocampus [39]. Glutamate is an excitatory neurotransmitter involved in synaptic transmission and is closely associated with learning and memory during aging. Learning and memory formation are associated with long-term potentiation (LTP), and maintenance of hippocampal synaptic LTP requires activation of glutamate receptors: N-methyl-D-aspartate receptors (NMDAR) [147]. The composition and function of NMDAR have been found to decrease with age in rodent models [148]; although NMDAR-mediated synaptic current dynamics remain unchanged in the CA1 region [149], the inactivation rate of these currents increases with aging [150]. Similarly, the expression of the postsynaptic glutamate receptors α-amino-3-hydroxy-5-methyl-4-isoxazole-propionicacid receptor (AMPAR) and its GluA2 subunit are also reduced in the CA1 region of aged mice [151], and AMPAR-mediated synaptic current strength has been shown to be diminished in aged mice [152]. Furthermore, the levels of many synapse-associated proteins, including synaptophysin, SNAP25 and PSD95, are lower in the hippocampi of aged rats [151, 153]. Indeed, the signaling of the inhibitory neurotransmitter gamma-aminobutyric acid (GABA) is equally impaired at aging synapses. McQuail et al. discovered that the aged hippocampus has a reduced frequency of inhibitory postsynaptic potentials driven by GABA, and a reduction in both current intensity and duration [154], thereby disrupting GABAergic interneurons and inhibitory synaptic circuits.

Additionally, a number of humoral indicators associated with brain aging have been identified. In both the blood and cerebrospinal fluid, high levels of CCL11 and 2-microglobulin have been shown to be related to diminished neurogenesis [155, 156]. The metallopeptidase inhibitor TIMP2 is associated with recovery of hippocampal neural activity [157]. The protein levels of TREM2, an immune signaling hub gene, increased progressively with age in the CSF, reflecting microglial function, and were also associated with AD [158]. In addition to changes in body fluids, the microglia of young and aged mice differed dramatically. Using transmission electron microscopy, Marschallinger et al. observed the cytoplasmic content of mice that were three and twenty months old. They observed that microglia in the aging hippocampus had distinct lipid droplets, but did not observe this in other cells [159]. Jin et al. identified a unique highly activated microglia (HAM) cell type that expressed an age-related activation pattern that triggers brain inflammation rather than the traditional M1/M2 pattern. In addition, Lpl and Lgals3 may be effective candidate markers for HAM [160].

## 6. Emerging Technologies and Approaches in Aging Biomarker Research

### 6.1. Omics technologies for comprehensive profiling

Aging is a continuous process that involves various detectable biomarker changes in the body and is influenced by the occurrence of diseases or decline of organ systems. Conventional methods of measuring aging rely on isolated indicators such as telomere length or ROS components, but these are insufficient to systematically and accurately describe aging. Owing to the rapid growth of omics technologies in recent years, aging can be characterized from a higher perspective using omics data. High-throughput sequencing, mass spectrometry (MS),and other quantitative techniques allow us to understand aging from the perspective of genes, tissue components, metabolites, and epigenetics. Multiple omics techniques can be integrated for aging identification to form a complex but accurate network structure [161].

*Genomics:* The process of aging is ongoing and regulated by numerous genes in the genome; however, we do not have a clear indicator to confirm that a gene is related to aging. Age-related genes may vary considerably among populations, and studies on a single population may overestimate the effects of a single gene. Therefore, studying a single gene might not capture the effects of aging on the entire genome. GWAS aims to determine the association between genotype and phenotype by detecting variations in allele frequencies of genetic variation between individuals with similar ancestry but different phenotypes [162]. Although SNP are the most frequently examined genetic variations in GWAS, copy number variation and sequence variation in the human genome have also been considered. GWAS tests the genomes of thousands of people for numerous genetic variations to identify those that are statistically linked to certain traits or disorders [163]. Therefore, genetic data from long-lived individuals in a population can provide insight into the longevity of genomes in specific populations. In particular, genes near the 5q33.3 locus of FOXO3 have been consistently associated with longevity. Timmers et al. analyzed genome-wide associations and mortality risk factor data from one million genotyped subjects with parental lifespan information using GWAS [164]. They replicated previously unconfirmed findings near FURIN/FES, CDKN2B-AS1, 13q21.31, PSORS1C3, ZW10, and ATXN2/BRAP, and discovered and validated new findings near IGF2R, ABO, and ZC3HC1. They also confirmed the roles of the 5q33.3/EBF1 and FOXO3 loci. Despite such extensive data, they affirmed that substantial differences in life expectancy at the population level were hard to achieve solely via genomic information. However, aging can be measured using life expectancy and health span, which are not equivalent when performing a GWAS. Zenin et al. identified 12 loci associated with human health span data that did not overlap with previous GWAS on lifespan [165].

Therefore, genomic analyses, especially GWAS, can identify biomarkers of differential gene expression associated with lifespan or health span. Analyzing these differential genes helps to better understand aging, while more refined comparison indicators cannot be equated with aging, and further research is required.

*Proteomics:* Proteins directly execute most biological activities. Compared to gene expression, proteins have more complex functional structures owing to post-translational modifications during transcription or translation, resulting in more complex aging effects. In traditional research fields, single biomarkers are used as indicators of aging, whereas proteomics can increase throughput to thousands or even tens of thousands simultaneously. Differential samples can be simultaneously scanned and observed using high-sensitivity MS and isotopic technology. This changes the incompleteness of the single-angle analysis and focuses more on system analysis from a system perspective.

Proteomic technology has been used from different perspectives to discuss aging, lifespan, and other related topics [166]. The age of the subjects ranged from newborns less than 1 year old to elderly people up to 95 years old, but the sample size varied greatly. In aging proteomics, samples are not limited to plasma or tissues. To date, various types of tissues, such as skeletal muscle, bone marrow, saliva, and urine, have been used for proteomic analysis. From a technical perspective, most studies have used LC-MS/MS to identify proteins and peptides. However, owing to the complex in vivo environment, unbiased quantitative detection of proteins remains challenging. Some studies have used the relatively advanced SOMAscan method developed by SomaLogic, which quantifies protein abundance in plasma using slow-off-rate-modified aptamers with high affinity for proteins. Owing to the length of this article, we will not analyze the data of existing research papers in the following summary, but focus on the summary of recent review papers. Ubaida et al. conducted a systematic review of several Google Scholar papers that used proteomics and aging as keywords [167]. They reanalyzed the data from these studies and identified 1,128 proteins that were reported as potential biomarkers in two or more studies. Interestingly, 751 proteins, including ANXA1 and HSPB1, were consistently detected in different groups using different tissues for proteomic analysis. Several potential biomarkers have been previously validated. Moreover, the authors summarized the common differential proteins reported in at least five papers and performed corresponding bioinformatics analyses and nonlinear and linear regression to propose models of aging. Moaddel et al. summarized and reanalyzed previous data using various proteomics and age-related keywords [168]. They collected proteomic data from subjects aged 14-103 years. Although LC-MS/MS is the most commonly used proteomics technology, they also included papers that used newer methods, such as the SOMAscan assay and Proximity Extension Assay (PEA O-Link). They identified 4,077 proteins in plasma data, of which 232 were highly correlated with age. These 232 differential proteins were used for further bioinformatics analysis, which revealed 112 potential age-related signaling pathways belonging to 21 signaling pathway categories.

Post-translational modifications (PTMs) of proteins can form more complex and diverse functional structures compared to gene expression. Moreover, PTMs are crucial for the aging process [169]. However, protein name-aging indicator regression analysis for PTM-type analysis remains scarce. Many bioinformatics analysis works and reviews, including Luigi Ferrucci’s work, summarize differently numbered proteins into single entries and then perform enrichment analysis. Moreover, in clinical diagnosis, PTM products are rarely used as markers. However, with the development of MS, omics technology can also identify different types of PTM protein content that change with age. For example, Collins et al. used data-independent acquisition (DIA) to achieve the unbiased quantitative detection of PTM proteins [170]. To date, several analysis software packages and corresponding product pipelines based on the DIA technology have been successfully developed.

In summary, according to existing proteomic data, aging is closely related to protein changes. Proteomics can provide indicators of aging. Analysis of deviation from the mean value of proteins may help in the diagnosis of diseases. This finding is clinically important. Another advantage of proteomics is that it can provide direct evidence of biomarkers that are important for disease diagnosis. PTM protein identification has a relatively short development time in proteomics; however, considering the importance of PTM in life activities, its role in aging research cannot be ignored.

*Metabolomics*: Metabolomics is an omics technology that uses MS or nuclear magnetic resonance to measure small molecules (< 1500 Da) in biological samples such as cerebrospinal fluid, saliva, muscle, and plasma. These molecules, known as metabolites, reflect the metabolic status and physiological functions of an organism. Metabolomics can reveal age-related changes in metabolism, which is one of the hallmarks of aging biology. Several platforms such as HMDB and MetaboAge have been established to explore the relationship between metabolomics and aging. Adav et al. summarized metabolomics and aging research from various perspectives [171]. They classified age-related metabolic changes into eight categories: lipid and lipoprotein metabolism, steroid hormones and menopause, amino acid metabolism, urinary system and excretion, carbohydrate metabolism, dietary changes, oxidative stress, and inflammatory responses. They also identified population-specific aging markers influenced by sex, race, health status, and age. Therefore, when studying the relationship between metabolomics and aging, the impact of multiple dimensions on metabolomics data must be considered. Moreover, some factors, such as sample collection and processing, may have affected the results. Metabolomics can identify meaningful markers of the aging process that are related to many diseases, such as coronary heart disease, metabolic syndrome, ischemic heart disease, obesity, type 2 diabetes, and other diseases that affect patients' quality of life and lifespan [172]. These findings have clinical importance for aging interventions and prevention. However, many challenges remain in this field, including the difficulty in identifying unknown metabolites. In the future, with improvements in the integration of multi-omics data and MS, more evidence can be provided to understand the complex aging process.

### 6.2. Machine learning and data mining approaches in biomarker discovery

In recent years, various machine learning approaches have been presented for estimating the BA. Previous omics studies have shown that a single biomarker is often insufficient to assess aging; however, the use of multiple biomarkers with different properties increases the complexity and cost of measurement. Therefore, a major research goal is to identify suitable biomarkers with potential research value. One approach is to use supervised multicomponent comparisons, linear regression, or PCA dimensionality reduction to obtain a set of biomarkers. Another approach is to use computer processing to obtain index weights or no-threshold decision making to extract more effective models with limited biomarkers. For example, simple random forest and DNN algorithms can significantly improve predictive performance [173]. These are the typical data analysis methods used in machine learning, and many biomarkers have been successfully identified using these methods. However, owing to the instability and complexity of biological systems, these data are highly complex. With recent advancements in artificial intelligence, machine learning and deep neural networks have enabled the fast analysis and extraction of critical data from complex systems and data. Altmann et al. studied the ranking of feature importance using the permutation feature importance score (PFI) method. Through multiple iterations, they calculated the average value of feature importance [174]. This method is effective and can be used to efficiently obtain important indicators. However, these indicators are not completely independent, and some related indicators are redundant as biomarkers. The PFI method did not provide any advantages as a biomarker. Therefore, appropriate evaluation methods should avoid data pollution, reduce the strong coupling effect between extraction indicators, and identify more independent biomarkers for predicting aging. In recent years, machine learning methods have been widely used to rationalize omics data and several representative ML applications have been developed, including RapidMiner, KINME, Weka, and Galaxy [175]. However, despite the rationalization of packaging, these programs lower the technical barriers for operators to some extent, but lack transparency and have limited adjustable parameters. Users must master computer languages, such as Python or R, to call machine learning or deep learning packages. Blood samples are good and viable tests for predicting age, and common blood tests can often assess a subject’s general health and detect the first signs of disease. Mamoshina et al. trained a model based on DNAm clock biomarkers to measure BA, using blood indicators as parameters [176]. Mamoshina et al. trained deep neural networks on population-specific datasets. Samples from Korean, Eastern European, and Canadian ethnicities were used. They trained the network within each group and tested it on a different test set with each available group. When trained and tested on the same population, the models showed good predictions for all three data sets. However, the accuracy values declined when tested in different ethnicities, and simply aggregating biomarkers into one model did not reflect all aspects of aging [176]. Holzscheck et al. used gene expression data as the input layer for a rationalized deep learning network that predicted BA based on the data [177]. After several cycles, the predicted age was positively correlated with the CA. The trained model could also predict various biological processes, such as the effect of virtual targeted knockouts on biological lifespan, the influence of various pathways on aging, and novel age-related biomarkers. This study provides an example of omics research using machine and deep learning. Biologists can use powerful AI models to discover a wider range of biomarkers from larger datasets.

BA estimation is the only example of machine learning that is used in aging research. Machine learning and artificial intelligence modules are generalized tools that make full use of deep learning networks such as transformers, CNN, and RNN, which can also be used to predict survival rates and brain-related BA. The key is to rationalize the relationship between the output and input indicators. However, some inherent disadvantages of machine learning also exist, such as the black box effect, which complicates the practical application of CA and BA; this comes not only from the measurement error of BA and CA, but also from the variation in indicators caused by different experimental conditions. Therefore, additional methods have been introduced to solve this problem. In short, with more data available under big data, BA can be better predicted through omics or a series of indicators.

**Table 2:** Summary of non-invasive imaging application in aging processes.

Imaging modality	Specific method	Imaging content	Imaging agents/ Biomarker change	Imaging scale	Reference
**PET**	Aβ-PET	Aβ plaques	^11^C-PiB	Brain	[182-185]
			^11^C-AZD2184		
			^18^F-florbetapir		
			^18^F-flutemetamol		
			^18^F-florbetaben		
	Tau-PET	Tau proteins	^18^F-AV1451	Brain	[186, 187]
	FDG-PET	Glucose metabolism	^18^F-fluorodeoxyglucose	Brain	[188-190]
	FDG-PET/CT	SUV; SMI; Volume	^18^F-fluorodeoxyglucose	Skeletal muscle	[191-193]
**Structural MRI**	T1WI	Brain volume	↓	Brain	[194, 195]
	T2-FLAIR	Water content	↑		
	DTI	Water diffusion	↑		
**Functional MRI**	MRS	Metabolite	↓	Brain	[200]
	PWI (ASL)	CBF	↓	Brain	[196]
	Rs-fMRI	Brain network connectivity (BOLD)	↓	Brain	[202, 203]
	Task-fMRI	Brain area activation	↓	Brain	[204]

Abbreviations: PET, positron emission tomography, MRI, magnetic resonance imaging, FDG-PET, fluorodeoxyglucose positron emission tomography , T1WI, T1-weighted imaging, T2-FLAIR, T2-fluid attenuated inversion recovery, DTI, diffusion tensor imaging, MRS, magnetic resonance spectroscopy, PWI, perfusion-weighted imaging, ASL, arterial spin labeling, Rs-fMRI, resting-state functional MRI, Task-fMRI, task functional MRI, SUV, standardized uptake value, SMI, skeletal muscle index, CBF, cerebral blood flow, BOLD, blood-oxygen level dependent

### 6.3. Imaging techniques for non-invasive assessment of aging processes

With the rapid development of both imaging and physical technologies, a wealth of imaging modalities have been widely used in clinical medicine. Molecular and functional alterations during the course of aging may be detected using a range of imaging techniques, such as computed tomography (CT), magnetic resonance imaging (MRI), ultrasound, PET emission tomography, and single-photon emission computed tomography (SPECT). In this section we focus on the use of PET and MRI imaging in the assessment of aging, and a detailed summary is presented in Table 2.

PET imaging, based on the distinctive identification of targets present in the body, not only directly reflects abnormalities in the early phase of disorders, but also offers unique advantages in revealing alterations in biomarkers of the aging process [178]. AD is an age-related neurodegeneration that mostly affects people of advanced age. AT(N) biomarkers are ones that the NIA-AA framework suggests using: markers of neurodegeneration (N), amyloid deposition (A), and neurofibrillary tangles Tau (T), that support or exclude the diagnosis of AD and brain aging [179]. Aβ and Tau PET imaging accomplish early visualization of disease primarily by quantitatively assessing the Aβ plaque deposition and Tau tangles in the living brain [180, 181]. Commonly used tracers for Aβ PET include^18^F-florbetaben, ^18^F-flutemetamol, ^11^C-PiB, ^18^F-florbetapir, which have received regulatory approval for use in medical facilities in the US [182, 183]. In addition, ^11^C-AZD2184 as an analog of ^11^C-PiB also showed considerably higher uptake in critical brain regions of AD patients than in normal brain tissue, with higher affinity and specificity for Aβ compared with ^11^C-PiB, and may have better results for diagnosing early AD [184, 185]. For tau PET imaging, ^18^F-AV1451 is the most researched tau-specific PET tracer, and the intracerebral distribution obtained is consistent with the known deposition of tau regions in neuropathological studies. Therefore, it can detect pathological tau proteins specific for AD to a large extent [186, 187]. Moreover, fluorodeoxyglucose positron emission tomography (FDG-PET) been reported to be a promising biomarker of synaptic activity for predicting AD progression [188]. When ^18^F-fluorodeoxyglucose uptake in the brain is reduced, as was measured using FDG-PET, it indicates a decrease in glucose metabolism and may respond to synaptic dysfunction [189]; for the AD signature hypometabolism, the cut point is 1.31 [190]. Sarcopenia, which commonly affects the elderly, plays a decisive role in confirming the diagnosis through estimation of muscle mass and volume using FDG-PET/CT. Generally, CT is commonly used to clinically observe muscle volume, whereas the metabolic information provided by FDG-PET can complement the anatomical correlation and characterization of the skeletal muscle obtained using CT [191]. Normal skeletal muscle shows uniform FDG uptake, with maximum standardized uptake values (SUVmax) usually between 0.5 and 2.2 [192]; comparatively, patients with sarcopenia may exhibit low metabolism and a reduction in muscle circumference [193].

In comparison to PET, MRI provides superior contrast for soft tissues, particularly the brain and abdomen. Consequently, the use of MRI in aging studies tends to focus on the process of brain aging. This demonstrates age-related changes in the structural makeup of the brain in vivo. Atrophy is one of the most important changes that occur during aging. This change may be tracked via measurements of the macroscopic dimensions of the brain and may also be seen as a localized loss of microscopic tissue, which manifests as an increase in the percentage of water in the brain in some instances [194, 195]. In addition to structural alterations, modifications in the brain function have been linked to aging and age-related neurodegenerative diseases. Functional magnetic resonance imaging (fMRI) can provide details on vascular and neural functions. Elderly people have a reduced volume of cerebral blood flow (CBF) across the whole brain [196], and this change in CBF with aging is linked to physiological shifts in the pressure of carbon dioxide in the arterial blood [197-199]. Magnetic Resonance Spectroscopy (MRS) uses a series of radiofrequency pulses to disturb the nuclei of various molecules and detect the resulting resonance signals. Because the frequency of a signal is determined by the precise chemical structure of the molecule, the levels of various metabolites in the brain can be calculated as an indirect assessment of aging [200].

Resting-state fMRI (rs-fMRI) measures brain network connectivity using blood-oxygen level-dependent (BOLD) signals, which can be applied to evaluate neurocognitive aging. Tau protein levels are corelated with greater functional connectivity in both normal elderly individuals and patients with AD [201]. However, another recent study suggested that decreased functional connectivity is associated with aging [202]. A meta-analysis showed that within the Default Mode Network (DMN), functional connection strength exhibits an inverse U-shape, reaching its peak in adulthood, after which is gradually declines [203]. Task-fMRI measures brain activaty during a specific cognitive task. Eevent-boundary-evoked brain activation in the hippocampus and posterior medial network decreases with age and can predict episodic memory ability [204]. MRI-derived brain age is a widely adopted biomarker of cognitive aging [205, 206]. The application of a deep learning algorithm makes the predicted brain age more accurate and reliable [207]. Critically, the deviation of individual brain ages from typical brain age trajectories can predict diseases such as schizophrenia [208] and mild cognitive impairment (MCI) [209].

Although MRI has been widely used in clinical practice, it mainly indirectly reflects aging-induced changes in the brain, and more methods are required to improve the imaging capability of MRI for biomarkers of aging.

## 7. Challenges and Limitations in Aging Biomarker Research

### 7.1. Variability and heterogeneity in aging trajectories

Complex life activities are characterized by substantial heterogeneity from individuals to cells. Aging is a necessary part of an individual’s life, and its high variability and heterogeneity are first reflected in gene transcription. Numerous age-related markers result from alterations in transcription, but the aging phenotype varies [210]. Based on various senescence inducers, cell types, and stages of senescence, the analysis of substantial sequencing data from human and mouse fibroblasts has revealed discrepancies in transcriptome profiles and SASPs [211]. In aged mice, p16 and p21 aggregate greater in hippocampal microglia, oligodendrocyte progenitor cells, and oligodendrocytes with some degree of heterogeneity [212]. The mouse senescent cell atlas, completed in 2020, contains sequencing data from 23 mouse tissues collected throughout their lifespan and demonstrates that p16, E2f2, Lmnb1, Tnf, and Itgax expression increases considerably with aging [213]; however, E2f2 is typically downregulated in senescent cells [214]. Another study compared transcriptome differences in the spleen, kidney, and lungs of aging and young mice using single-cell sequencing and discovered that different cell types display different aging trajectories as a consequence of gene enrichment [215]. In conclusion, in most in vitro experiments, senescent cells were dispersed in different cell masses rather than forming specific clusters, further demonstrating the existence of heterogeneity. A clinical study revealed that although certain serum biomarkers are associated with in vivo phenotypes (e.g., hippocampal volume and cognitive performance), they are not associated with CA [91]. This supports the view that the difference between CA and BA increases owing to various susceptibility factors, leading to increased heterogeneity in the elderly population. Aging process also exhibits substantial heterogeneity in terms of sex. For example, healthy women have 41%, 50%, and 39% lower risks than healthy men, for developing cancer, cardiac diseases, or death, respectively. Following cancer incidence, men were found to be twice as likely as women to develop disability or dementia; following the onset of disability or dementia, men showed twice the mortality rate compared to women. A higher percentage of women aged 75 and 85 years remained healthy compared with men of the same age [216]. Although age is highly associated with accelerated disease progression, inter-individual differences remain.

### 7.2. Standardization and reproducibility issues in biomarker assessment

Although important advances have been made in identifying biomarkers of aging, data from promising marker studies have been found not to be reproducible. The validation of a biomarker through repeated independent experiments improves the chances of establishing the authenticity of this marker and further demonstrates the consistency of the experimentally detected data using the intended target. Studies related to biomarkers with poorly reproducible results can waste time and effort and prove costly for other follow-up investigators attempting to reproduce the results. Many potential factors can contribute to low reproducibility, such as cohort-related factors, experimental conditions, biological variability of the marker, statistical methodological flaws, and a lack of appropriate validation methods, which usually introduce random errors or systematic biases, leading to inaccurate measurement results. For example, some forms of subject recruitment that use selection or exclusion criteria are less reproducible than random recruitment [217]. Meanwhile, the operability of the detection method, sensitivity of detection instrument, stability of detection reagents, pre-detection sampling, and post-detection preservation may be affected by systematic errors that interfere with the reproducibility of the data. Therefore, all subjects, whether in the experimental or control group, were required to perform at the same location and time period and use the same standardized procedure, which improved both the standardization and reproducibility of the experimental process. Variability in laboratory measurement procedures has been a major challenge in assessing biomarkers, and with the development of international protocols for standardized methods, we know that a complete set of standardized procedures for biomarker assessment includes not only a standardized measurement process, but also the way in which the measurement results are applied, including how to combine them with clinical information for diagnosis [218]. Therefore, the discovery of candidate biomarkers for clinical testing is a long and difficult process; however, the standardized assessment of aging biomarkers is still not fully universal and the establishment of a coherent and comprehensive standardized assessment procedure for aging biomarkers needs to be urgently addressed.

### 7.3. Ethical considerations and privacy concerns in biomarker utilization

Aging research brings various benefits to human health and society. However, as in other innovative biomedical fields, basic research on aging biomarkers and their clinical translation is usually conducted in animals and humans, which brings potential social and ethical risks. An ethical framework for clinical research is proposed based on major guidelines, statements, and other documents related to human research: value, scientific validity, good risk-benefit proportion, fair subject choice, independent evaluation, informed consent, and respect of subject’s interests [219]. Owing to the variability and heterogeneity of aging trajectories, which diminish the predictive value of aging biomarkers to some extent, their clinical validity is readily questioned, together with the absence of corresponding clinical therapies, which makes confirming their clinical efficacy harder. For physicians, a conclusive diagnosis based on test findings is challenging, and may include biases. Furthermore, the results must be communicated to the subjects without alarming them or diminishing the credibility of the test. Researchers will have to establish a manner of effectively and thoroughly educating individuals on the possible advantages and hazards of aging biomarker testing. The Belmont Report identified three principles: respect for persons, beneficence, and justice [220]. Respect for a person requires not deceiving the subject in any way and ensuring his or her autonomy; therefore, the subject should understand what tests are to be performed, why, and consent to any laboratory tests performed. In addition, aging biomarker research and the accompanying clinical trials raise the issue of whether the data should be made public to the subjects [221]. While subjects may benefit from early illness care and life planning, which might increase their quality of life, disclosure is likely to induce psychosocial discomfort because of the restricted availability of aging interventions, which may be detrimental to the subject’s quality of life. For example, those who are assessed as being at high risk for age-related diseases are likely to face discrimination [222]. In short, testing researcher’s abilities has become a complex and long-term challenge. Therefore, establishing a standardized ethical framework for aging research can guide researchers and clinicians. Adhering to the ethical principles of aging research will safeguard the long-term health of the field.

## 8. Applications and Potential of Aging Biomarkers

### 8.1. Early detection and prediction of age-related diseases

The discovery of aging biomarkers is helpful for early monitoring and prediction of age-related diseases. Jin et al. reviewed the role of aging biomarkers in the pathogenesis of AD, which is mainly caused by age-related factors [9]. Heterogeneous biomarkers such as DNAm and histone modifications can predict early disease risk and mortality [223]. A novel pharmacological target for AD therapy is the age-dependent gene regulatory network, a molecular network of elements that changes dynamically with aging. This network may be responsible for AD [224]. A BA algorithm based on aging biomarkers trained by Julia et al. in an older age cohort predicted an increased risk of all major diseases associated with aging, including dementia associated with accelerated aging [225]. Julia et al. incorporated the neurofilament light chain (NfL), the best predictor of neurological prognosis [226], into a BA algorithm, further improving the predictions of increased dementia risk without adding value, suggesting that aging biomarkers may be highly sensitive in predicting age-related diseases [225].

### 8.2. Monitoring interventions and assessing treatment efficacy

A thorough understanding of the multifactorial aging biomarkers underpins the need for an all-encompassing strategy for older patients, particularly the frail multimorbid elderly, to create a patient-centered and goal-oriented clinical management plan [227]. In clinical studies, biomarkers of aging can be used to identify individuals who are suitable and likely to be most receptive to aging-biology-focused therapies. In this type of trial, biomarkers can be used to validate target contacts or as surrogate endpoints for information that may change before clinical outcomes (e.g., frailty measurement [228]). For example, certain interventions can improve brain health by increasing the expression of biomarkers that delay brain aging. Timely monitoring of relevant indicators during the intervention process can help assess the corresponding treatment effects and subsequently adjust the treatment strategy [229]. In frail elderly patients, high IL-6 and low albumin levels are significantly associated with 3-month all-cause mortality, and measuring their expression may help provide tailored therapeutic interventions to reduce short-term mortality in hospitalized elderly patients [230]. In older adults undergoing surgery, age-related biomarkers have been shown to predict unfavorable outcomes such as surgical complications and hospital readmission [231].

### 8.3. Personalized medicine and targeted interventions for healthy aging

Healthy aging is an important topic in global aging research. The objective was to investigate and lengthen the healthy life expectancy, while reducing periods of disease and dysfunction [12]. From a medical and prevention perspective, researchers have focused on changes in individual aging, with the aim of screening high-risk groups with prompt intervention to achieve healthy aging. Bai et al. confirmed that, when measuring individual aging, BA determined using the BA equation according to the development of aging biomarkers is preferable to CA and aids in identifying people vulnerable to age-related disorders [232]. Bai et al. also screened seven types of aging biomarkers, and conducted BA evaluation on 852 healthy individuals, which were split into three groups based on age: normal, early, and delayed aging; they observed substantial differences in biomarkers in the four age groups [232]. Chu et al. found that extracellular vesicle treatment prolonged the lifespan of aged mice owing to decreased expression of the transcription factor BTB and CNC homolog 1 (Bach1) and enhanced expression of Nrf2. They suggested that targeting the Nrf2/Bach1 axis might repair oxidative stress damage in aged mice [233]. These findings indicate that aging biomarkers can contribute to the timely detection and intervention of individual aging and even may help delay aging.

Potential AD treatment candidates, such as DNMTs, Sirtuin 1 (SIRT1), and methyl-CpG-binding protein 2 (MeCP2), have been proposed following the exploration of a dynamic network that coordinates DNAm and histone modifications [234]. This suggests that aging biomarkers may be helpful in personalized medicine and targeted interventions for healthy aging. For instance, the multidimensional prognostic index (MPI), an indicator derived from a comprehensive geriatric assessment (CGA), uses information from eight domains relevant to the overall evaluation of older adults (mobility, nutrition, functional and cognitive status, pressure ulcer risk, multiple diseases, multi-medication treatment, and cohabitation) to estimate the overall risk of multidimensional impairment and provide a reliable prognostic measure. It has decent calibration, accuracy, and discrimination, and serves as a crucial point of reference for decision-making and resource allocation in clinical practice and research [227]. Combined with the detection of aging biomarkers, this study is expected to provide accurate and personalized care for patients and older adults.

## 9. Future Directions and Research Priorities

### 9.1. Integration of multi-modal biomarker approaches for comprehensive assessments

In the future, age-related assessments and interventions will require several assessment systems that integrate existing biomarkers. Bai et al. proposed that multi-model aging biomarker systems can be integrated and optimized through prospective cohort studies to estimate the probability of death and onset of age-related diseases. In particular, a streamlined aging evaluation technique was developed for primary care facilities [12]. Moreover, given the nonlinear and heterogeneous characteristics of age-related changes, the likelihood of a single biomarker meeting the ideal predictive or diagnostic criteria is very low. Because “omics” approaches are now widely available, potential biomarkers such as genes, non-coding RNAs, proteins, transcripts, and metabolites, can be identified without making any assumptions [235]. Combining a hypothesis-driven approach from a single biological pathway with a multi-omics approach from a physiological perspective may be a major challenge for future healthy aging biomarker research. Therefore, artificial intelligence must be introduced into research so that the complex interactions and network changes of markers can be integrated at the molecular, cellular, and bodily levels. This can help us to better comprehend the mechanisms underlying systemic aging and incorporate a single, seamless end-to-end approach for biomarker and drug discovery, target identification, and other processes that may speed up research and development procedures [236]. Although the use of neural networks in aging research is still in its infancy, neural networks have attracted the interest of an increasing number of biomedical researchers. The first cloud-based deep neural network (DNN) was approved by the Food and Drug Administration (FDA) in the medical device category. The FDA has also approved the creation of the first fully open-access database for AI/machine learning (ML)-based medical technologies, which will be continuously updated [237]. The FDA has authorized 178 medical devices with AI/ML capabilities as of October 5, 2022 [238]. While many privacy issues may be involved in the acquisition, generation, and use of health data, the guarantee established by the General Data Protection Regulation (GDPR) in Europe is that medical records will be used and transferred properly. Hopefully, they will not hinder meaningful technological development.

### 9.2. Longitudinal studies to capture dynamic changes in aging biomarkers

Notably, cross-sectional studies constitute the majority of existing research on the connection between aging and biomarkers. Therefore, longitudinal studies are necessary to periodically assess physical and mental functions across a range of ages and health situations [239]. A greater understanding of the rate of biological aging is promised by longitudinal research, which may also reveal, in the context of clinical trials, the extent to which the molecular and cellular effects of aging can be reduced or even reversed. In particular, cohorts of middle-aged participants will aid in the discovery of early biomarkers of healthy aging that may be overlooked in older adult research owing to selection bias. Dynamic variations in potential biomarkers can be studied through longitudinal examinations, which will provide more room for discussion on the biological process of aging and its heterogeneity among individuals [240, 241].

### 9.3. Translational potential and implementation challenges in clinical practice of aging

Translating basic research findings into clinical applications poses several challenges. For the development and validation of biomarkers, we must use animal models that age faster than humans and can be subjected to controlled modification of biological pathways or environmental factors. Budding yeast (*Saccharomyces cerevisiae*), fruit flies (*Drosophila melanogaster*), fish, laboratory mice (*Mus musculus*), and nematodes (*Caenorhabditis elegans*) are traditional animal models used in aging research [242]. Several conserved genes that regulate lifespan across long evolutionary distances have been identified in numerous studies using animal models [243]. African killifish, for example, age more quickly than mice and are therefore excellent models [244]. As models for human aging, each of these species has drawbacks and advantages; the similarities and differences in physiology, longevity, and aging characteristics must be considered. Therefore, the gap between laboratory conditions and real-life situations must be bridged [239]. For clinical applications, systematic and individualized assessment, and stratification of the elderly in hospitals and community health centers at all levels can help achieve the precise prevention and targeted management of age-related diseases. Effective use of limited health resources will be the focus of future research [12] (Table 3).

**Table 3:** Benefits and limits of monitoring aging biomarkers.

Title	Contents
**Benefits**	In-depth understanding of the formation processes and influencing factors of various aging phenotypes
	Comprehensive understanding of the effects on the structure and function of the human body with aging
	Early detection and prediction of age-related diseases
	Monitoring interventions and assessing treatment efficacy
	Personalized medicine and targeted interventions for healthy aging
**Limits**	Aging heterogeneity undermines some of the predictive value of biomarkers
	Lack of corresponding therapies makes the clinical validity of biomarkers questionable
	Inability to accurately diagnose disease based on the results of biomarkers
	Publicizing results maybe have an impact on the state of social life of patients

## 10. Conclusion

In this paper, we review the current advances in the field of aging biology and geriatrics, focusing on molecular, cellular, and organismal biomarkers and targets of aging. We highlighted the challenges and opportunities for identifying and validating reliable and comprehensive measures of the health span and BA. We also discuss the importance of considering the effects of individual aging biomarkers at distinct biological organization levels and the integration of multi-dimensional and multi-domain methods for studying the underlying processes of aging. We have emphasized that no single biomarker can encapsulate the complexity and heterogeneity of aging, and that multi-omics approaches may offer unbiased and systematic ways to discover new aging biomarkers. However, we acknowledge that these methods require further development and validation, in addition to ethical and privacy considerations surrounding data utilization. This review has some limitations, such as the omission of some relevant phenomena in aging biology, including autophagy and the microbiome. We suggest that future research should explore these topics in more depth and detail, and their potential roles as biomarkers or targets for healthy aging. We hope this review will stimulate further research and innovation in this exciting and rapidly evolving field.
